# Towards A Deeper Understanding of the Interfacial Adsorption of Enzyme Molecules in Gigaporous Polymeric Microspheres

**DOI:** 10.3390/polym8040116

**Published:** 2016-04-07

**Authors:** Weichen Wang, Weiqing Zhou, Wei Wei, Juan Li, Dongxia Hao, Zhiguo Su, Guanghui Ma

**Affiliations:** 1 National Key Laboratory of Biochemical Engineering, Institute of Process Engineering, Chinese Academy of Sciences, Beijing 100190, China; wchwang@ipe.ac.cn (W.Wa.); wqzhou@ipe.ac.cn (W.Z.); weiwei@ipe.ac.cn (W.We.); lijuan@ipe.ac.cn (J.L.); dxhao@ipe.ac.cn (D.H.); zgsu@ipe.ac.cn (Z.S.); 2 University of Chinese Academy of Sciences, Beijing 100049, China

**Keywords:** lipase, adsorption, gigaporous, mesoporous, microsphere, QCM-D

## Abstract

Compared with the one immobilized in the conventional mesoporous microspheres, the enzyme immobilized in gigaporous microspheres showed much higher activity and better stability. To gain a deeper understanding, we herein selected lipase as a prototype to comparatively analyze the adsorption behavior of lipase at interfaces in gigaporous and mesoporous polystyrene microspheres at very low lipase concentration, and further compared with the adsorption on a completely flat surface (a chip). Owing to the limited space of narrow pores, lipase molecules were inclined to be adsorbed as a monolayer in mesoporous microspheres. During this process, the interaction between lipase molecules and the interface was stronger, which could result in the structural change of lipase molecular and compromised specific activity. In addition to monolayer adsorption, more multilayer adsorption of enzyme molecules also occurred in gigaporous microspheres. Besides the adsorption state, the pore curvature also affected the lipase adsorption. Due to the multilayer adsorption, the excellent mass transfer properties for the substrate and the product in the large pores, and the small pore curvature, lipase immobilized in gigaporous microspheres showed better behaviors.

## 1. Introduction

Immobilization is one of the most effective approaches to improve the catalytic performance of enzymes. To gain a better performance, the immobilized carriers should be rationally designed and selected. It has been demonstrated that the physical properties of carriers, such as surface morphology [1,2], particle size [3,4], and inner porous structures [5,6,7,8] have an important impact on enzyme immobilization. Recently, many porous particles have also exhibited their distinctive performance as immobilized carriers [9,10]. It is worth noting that the pore size of these conventional carriers usually ranges from a few nanometers to tens of nanometers [11,12]. In this case, the enzyme penetration in most studies might be limited by the tiny pore size. The effect of pore size on the activity of enzymes has been a controversial issue. Because of the complexity of enzymes, different or even opposite results have been reported [13,14,15,16].

In our previous work, lipase was immobilized in polystyrene (PST) microspheres with different pore sizes of 14 nm (mesoporous), 100 nm (macroporous), and 300 nm (gigaporous). Fortunately, we found the specific activity of lipase immobilized in gigaporous microspheres was 2.87 times and 1.46 times more than that of mesoporous ones and the free lipase, respectively. In addition, the reusability and the stability of lipase in gigaporous microspheres was improved dramatically compared with those of mesoporous ones [17]. The significance of pore size has been demonstrated in this comparative investigation. Therefore, the reasons of these results and the detailed analysis of enzyme adsorption needed to be researched. There were many reports about the enzyme adsorbed in macroporous microspheres. Gross *et al.* studied the distribution and the molecular structure of lipase adsorbed in microspheres with different pore sizes. The average pore sizes of the carriers were usually 40–100 nm. The results showed that the enzyme is localized in an external shell of the bead [18,19,20,21]. They noticed the influences of pore size on the diffusion—the higher the pores, the better the activity due to the diffusion—however, they did not specialize the adsorption state. In addition, although macroporous microspheres were frequently used in enzyme adsorption, many studies mainly focused on the microspheres with a maximum pore size of 100 nm, which may be restricted by the preparation technology of microspheres. As for other researches about gigaporous microspheres, Miletić *et al.* investigated the poly(GMA-*co*-EGDMA) [poly(glycidyl methacrylate-*co*-ethylene glycol dimethacrylate)] microspheres with large pore size (over 300 nm) as the immobilized carriers of lipase. The main conclusions mostly focused on the immobilization and catalysis results [22], yet the effects of the adsorption state were not taken into consideration.

In this work, lipase B from *Candida antarctica* was selected as the prototype because of its wide applications. The adsorption time and the initial concentration of lipase were tailored to investigate the adsorption amount and the enzyme activity of lipase in the microspheres more precisely. Moreover, considering the possible effect of the pore curvature, lipase adsorption in microspheres was compared with that on the flat surface. In order to attain the real-time data of lipase adsorbed on flat surface, quartz crystal microbalance with dissipation monitoring (QCM-D) was used to monitor the adsorption process of enzyme molecules on flat surface [23].

The work carried out on the behaviors of lipase adsorption in the gigapores, in the mesopores, and on the flat surface, which was expected to provide a model for the enzyme immobilization in gigaporous microspheres.

## 2. Materials and Methods

### 2.1. Materials

Lipase B from *Candida antarctica* (CALB) (3.8 U/mg by activity assay) was kindly provided by Novozymes (Bagsværd, Denmark). The substrate *p*-nitrophenyl palmitate (*p*-NPP) of analytical grade was purchased from Sigma-Aldrich (St. Louis, MO, USA). The standard *p*-nitrophenol (*p*-NP) of analytical grade was purchased from Sinopharm Chemical Reagent Beijing Co., Ltd. (Beijing, China). The PST chips were purchased from Biolin Scientific AB (Goteborg, Sweden). The giga-/meso-porous PST microspheres were prepared by National Engineering Research Center of Biotechnology (Beijing, China). All chemicals were of analytical grade and used without further purification unless otherwise described.

**Figure 1:**
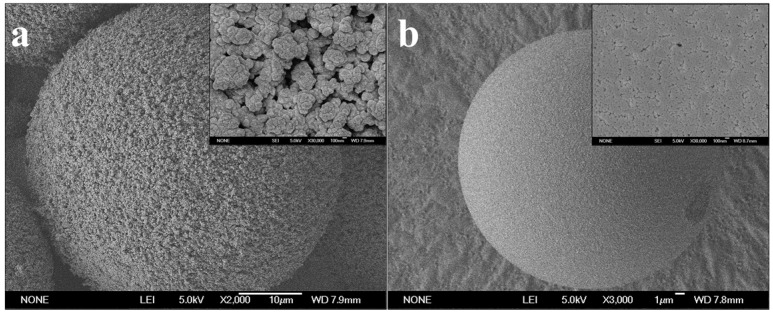
SEM images of PST-300 (**a**) and PST-14 (**b**) microspheres.

The scanning electron microscope (SEM, JEOL, JSM-6700F, Tokyo, Japan) images of the microspheres were shown as Figure 1. The structural data of the microspheres measured by mercury porosimetry (Micromeritics, AutoPore IV 9500, Norcross, GA, USA) were shown in Table 1. The gigaporous PST microspheres were recorded as PST-300, and the mesoporous ones were PST-14.

### 2.2. Adsorption of Lipase in PST Microspheres

Several groups of giga-/meso-porous PST microspheres (0.01 g) were added into the lipase solution (0.05 mg/mL, 2 mL). Then, the mixture was shaken gently at 25 °C. The adsorption time was 1, 2, 5, 8, 10, 20, 30 min, respectively. When the adsorption time was up to the pre-set value, the corresponding sample was taken out and centrifuged three times to separate the microspheres and the solutions (10,000 rpm, 3 min), and the lipase that was not adsorbed was washed out.

### 2.3. Assay of Adsorption Mass and Activity of Lipase in PST Microspheres

The amount of lipase adsorbed in microspheres was measured via the bicinchoninic acid (BCA) method at 562 nm using multimode microplate readers (Tecan, Infinite 200, Zurich, Switzerland). The protein concentration of initial lipase solution and residual supernatant were analyzed to determine the adsorption mass. The standard work agent was prepared by the mixture of BCA agent and Cu agent (50:1, *v*/*v*). A calibration curve was constructed from BSA solutions of known concentration (31.25–2000 μg/mL) and used to calculate the protein amount in initial and washing solutions [24].

The activity of lipase adsorbed in microspheres was also assayed by *p*-NPP hydrolysis. *p*-NPP can be hydrolyzed by lipase to *p*-NP. Firstly, the *p*-NP standard solution was prepared, and the calibration curve was the result. *p*-NPP was dissolved in acetone and then diluted with phosphate buffer (50 mmol/L, pH 7.0) containing Triton X-100 (1.25%, *w*/*v*). The *p*-NPP and immobilized lipase were mixed in a centrifuge tube with cap for 10 min at 37 °C. Then, the absorbance was monitored at 410 nm using multimode microplate readers.

### 2.4. CLSM Analysis of Lipase Adsorbed in Microspheres

Confocal laser scanning microscopy (CLSM) (Leica, TCS SP5, Wetzlar, Germany) was employed to investigate the distribution of lipase adsorbed in the microspheres at the time point of 2, 8, 30, 40 min. Fluorescamine was used as a reagent for the detection of lipase, which was soluble in acetone at 50 mg/mL. The fluorescamine solution was added to react with PST-lipase for 3 min. The samples were excited at 390 nm, and the fluorescent images at 480 nm wavelengths were then obtained.

### 2.5. QCM-D Measurement

Phosphate buffer was filtered through membrane of 0.22 μm and then ultrasonic degassed for 20 min. The QCM-D (Q-SENSE E4, Biolin Scientific AB, Goteborg, Sweden) measurement was conducted at 37 °C, and PST chip was cast into the measure chamber. Phosphate buffer was injected into the chamber. When the baseline was stabilized, a diluted lipase solution was injected into the chamber using a flow rate of 50 μL/min. This process simulated the immobilization procedure. Adsorption time of lipase on PST chip was 90 min from the optimized results of pre-experiments. Then, the chip was rinsed by the phosphate buffer, and the desorbed lipase molecules were rinsed out. When the baseline was stabilized again, *p*-NPP was injected into the chamber to simulate the hydrolysis reaction of *p*-NPP catalyzed by the immobilized lipase. The *p*-NPP mixture (0.05 mg/mL) comprised *p*-NPP (0.05 mg), acetone (12.5 μL), and phosphate buffer (987.5 μL, 0.01 mol/L, pH 7.2).

The determined parameters were Δ*f* and Δ*D*. Δ*f* represented the frequency change on the chip surface, which reflected the change of adsorption mass. Δ*D* represented the change of dissipation factors, which reflected the change of adsorption viscoelastic. The |Δ*D*/Δ*f*| ratio reflected the adsorption tightness, which was contrariwise proportional to the adsorption tightness. A large |Δ*D*/Δ*f*| ratio indicated an extended structure or loose binding between the interacting molecules [25].

At the same time, the adsorption and the catalysis process was monitored, as a function over time, by simultaneously recording the shifts in the frequency (Δ*f*) and in the energy dissipation (Δ*D*) at the fundamental resonant frequency, along with the third, fifth, and seventh overtones, until the adsorption reached a steady-state. At this time, the long-term stability of the frequency was within 1 Hz, which was negligible when compared with the frequency shifts caused by adsorption. Normalized data obtained from different overtones were used in the calculation of adsorption mass, thickness, and viscoelastic properties of adsorbed layers using the Voigt model. Sauerbrey mass was calculated using the Sauerbrey equation [26]:

(1) M=−​  ​(C /n) Δf

where Δ*f*, *M*, and *n* represented frequency change, adsorbed mass per unit area, and overtone number, respectively. C was the mass sensitivity constant (17.7 ng/cm^2^ Hz). The adsorption mass was inversely proportional to the Δ*f* value.

### 2.6. Assay of Adsorption Mass and Activity of Lipase on PST Chip

The adsorption mass of lipase on PST chip can be calculated by Equation (1), of which the unit was ng/cm^2^. The activity can be calculated by the decreased amount of the substrate *p*-NPP. The activity unit (U) of lipase was defined as the amount of lipase required to hydrolyze 1 μmol of *p*-NPP per minute at 37 °C (pH 7.0). Then, the lipase activity was calculated by:

(2) $$u=\frac{(M_{1}-M_{2})\times A_{\mathrm{chip}}\times 10^{6}}{377.52\times t\times M_{L}}$$

where *M*_1_ was the initial adsorption mass of *p*-NPP per unit area, *M*_2_ was the adsorption mass of *p*-NPP per unit area after hydrolysis, A_chip_ was the area of PST chip, of which the value was 1.54 cm^2^, the value 377.52 was the molecular weight of *p*-NPP, *M*_L_ was the lipase dosage, and *t* was the reaction time.

In addition, the specific activity of lipase was defined as the activity of an enzyme per milligram of total protein (U/mg protein).

## 3. Results and Discussion

### 3.1. Analysis of Lipase Adsorption in PST Microspheres

**Figure 2:**
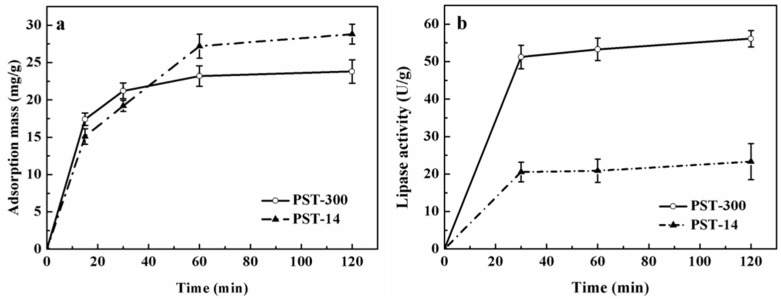
Adsorption process of lipase (0.05 mg/mL) in PST microspheres within 120 min ((**a**) Adsorption mass; (**b**) Lipase activity).

Lipase immobilized in the gigaporous PST microspheres showed higher activity, stability, reusability, and better kinetic performance than that in the mesoporous PST ones. In order to attain the detailed adsorption behaviors of lipase, PST-300 and PST-14 were used for the real-time adsorption of lipase. Figure 2 showed the adsorption process of lipase in PST-300 and PST-14, including the change of adsorption mass and lipase activity within 120 min. It can be seen from Figure 2a that the adsorption reached equilibrium for lipase in PST-300 after the time point of 30 min and for lipase in PST-14 after the time point of 60 min. From Figure 2b, it can be seen that the initial phase of lipase adsorption (before the time point of 30 min) had great influences on lipase activity.

**Figure 3:**
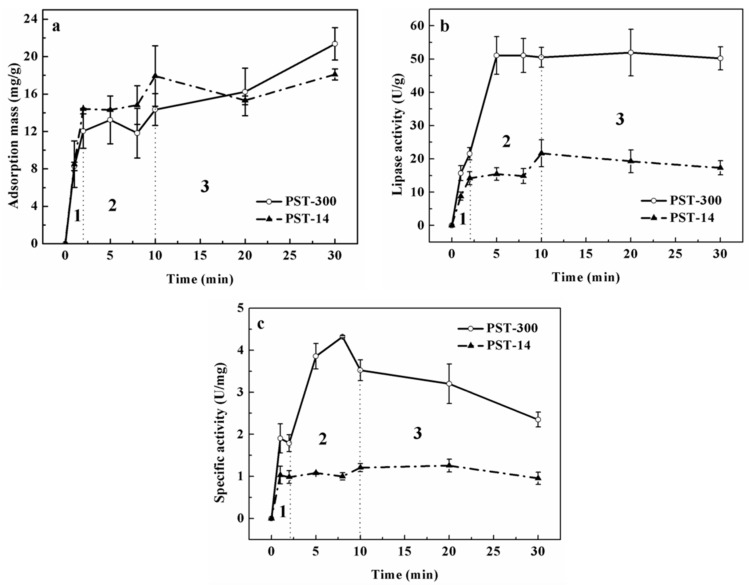
Adsorption of lipase (0.05 mg/mL) into PST microspheres within short time ((**a**) Adsorption mass; (**b**) Lipase activity; (**c**) Specific activity). Specific activity of free CALB: 3.8 U/mg.

In order to study the adsorption of lipase more elaborately, the experiments were carried out firstly under the condition of low lipase concentration (0.05 mg/mL) and short adsorption time (within 30 min). The adsorption states of lipase in gigaporous and mesoporous microspheres were analyzed comparatively. The changes of adsorption mass, enzyme activity, and specific activity of lipase in PST microspheres with adsorption time are shown as Figure 3, which shows a clear difference between the two types of microspheres.

**Figure 4:**
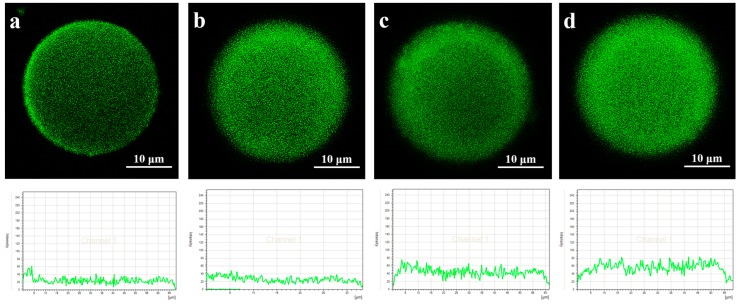
CLSM images and fluorescence distribution of lipase adsorbed in gigaporous microspheres at different time point ((**a**) 2 min; (**b**) 8 min; (**c**) 30 min; (**d**) 40 min).

**Figure 5:**
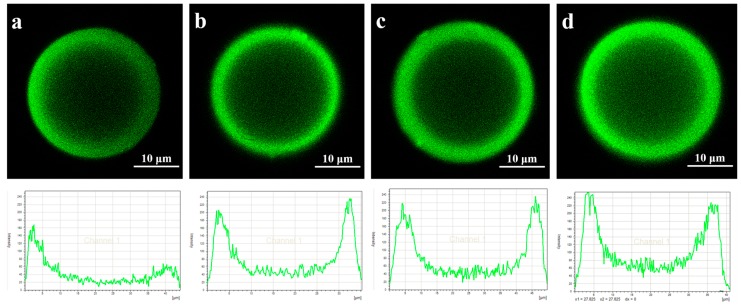
CLSM images and fluorescence distribution of lipase adsorbed in mesoporous microspheres at different time point ((**a**) 2 min; (**b**) 8 min; (**c**) 30 min; (**d**) 40 min).

In Figure 3a, the adsorption mass increased rapidly in the first two minutes, and monolayer adsorption mainly occurred in this stage since the enzyme concentration was very low (0.05 mg/mL). For the gigaporous microspheres, the adsorption mass basically maintained a sustained growth from the time point of 2 to 30 min. As for mesoporous microspheres, the adsorption mass increased from 12.2 to 15.6 mg/g (from the time point of 2 to 10 min), and nearly kept stable after the time point of 10 min. Therefore, after the time point of 10 min, the adsorption was mainly multilayer. Figure 4 and Figure 5 were the CLSM images and the change of fluorescence intensity of lipase adsorbed in gigaporous and mesoporous microspheres with the adsorption time. For the gigaporous particles, before the time point of 2 min, lipase molecules entered the whole particle and dispersed homogeneously (Figure 4a), while, in the mesoporous particles, most enzymes adsorbed in the shell (Figure 5a). Consistent with the increase of adsorption mass in Figure 3a, after the time point of 2 min, the fluorescence intensity in gigaporous particles exhibited a stable and uniform enhance (Figure 4b–d). As for lipase adsorbed in the mesoporous microspheres, only the fluorescence intensity of shell increased due to the limit of a smaller pore size (Figure 5b–d).

The effects of the different adsorption behavior in the carriers with various porous structures were researched. The dramatic difference between their enzyme activities (Figure 3b), especially the specific activities (Figure 3c) gave very interesting results. In the first stage (before the time point of 2 min), the specific activity of lipase immobilized in the gigaporous particles was 1.79 U/mg, and that in the mesoporous particles was only 0.99 U/mg. According to the fluorescence distribution, the specific area of the gigaporous and mesoporous particles, and the area per lipase molecule occupied in ideal state, we calculated the amount of enzyme adsorbed on the unit area of the microspheres, 5.34 × 10^11^ and 3.12 × 10^10^, respectively. The amount is far less than the molecule number needed for monolayer adsorption in both type of particles (7.85 × 10^14^, PST-300; 7.68 × 10^15^, PST-14, 1/10 diameter). Therefore, it tended to be monolayer adsorption in this stage theoretically. Since both kinds of microsphere were based on P(ST-DVB) [poly(styrene-divinylbenzene)], which allowed physical adsorption of lipase, the only difference is the curvature of pores in the stage of monolayer adsorption. The curvature of gigapores was much lower, and the surface of gigapores was flatter than that of mesopores. According to the effect of curvature to proteins [27], when the value of curvature was bigger, the contact area and the deformation were larger. Additionally, the structure of the enzyme molecules cannot be well maintained. Conversely, the flatter surface was beneficial for retaining the molecular structure. This explained the difference of specific activity in gigaporous and mesoporous particles before the time point of 2 min.

For lipase immobilized in gigaporous particles, from the time point of 2 to 8 min in Figure 3c, their specific activity increased rapidly to 3.86 U/mg. While the specific activity of lipase immobilized in mesoporous particles did not have significant change. The main reason of the difference is also due to the pore structure. From the time point of 2 min, multilayer adsorption occurred in gigapores with the large enough space. Multilayer adsorption effectively maintained the enzyme activity. The adsorption of lipase in mesoporous microspheres was still inclined to be monolayer adsorption because the mesopores restricted the distribution of lipase molecules (the pore size is 14.7 nm; the molecular size of CALB is 6.92 × 5.05 × 8.67 nm^3^ [28]). Since the monolayer adsorption of lipase was dominant in mesoporous microspheres, the enzyme activity and the specific activity were obviously lower than those in the gigaporous ones.

After the time point of 8 min, though the adsorption mass still increased in the gigaporous microspheres, the enzyme activity had little change, and the specific activity decreased. This was presumably due to the fact that with more enzyme adsorbed in the pores, the cross-section of the pores might be reduced; thus, the diffusion resistance for the substrate and the product was increased [29].

### 3.2. Comparison of the Adsorption in Microsphere with the Adsorption on Flat Surface

**Figure 6:**
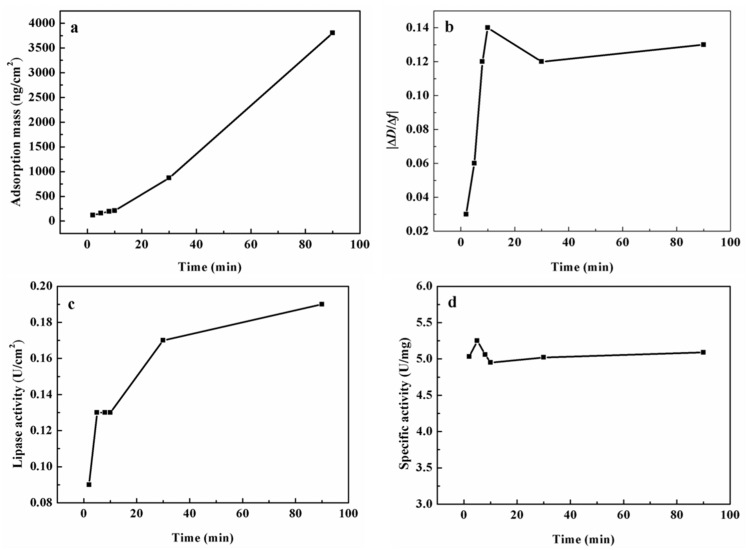
Adsorption of lipase on PST chip determined by QCM-D ((**a**) Adsorption mass; (**b**) |Δ*D*/Δ*f*| ratio; (**c**) Lipase activity; (**d**) Specific activity).

The reasons for high enzyme activity and specific activity of lipase in gigaporous microspheres are explained by the difference of the curvature and the monolayer/multilayer adsorption. The adsorption on a flat surface was further compared to find what happened when there was no limit of pores. Quartz crystal microbalance with dissipation monitoring (QCM-D) technology was adopted to investigate the adsorption behavior of lipase on PST chips and the influence on enzyme activity. Figure 6 showed the adsorption parameters of lipase on PST chips.

From Figure 6a, we could see that the increase of adsorption mass was a rough linear rise on the chip when excluding the influence of diffusion transfer in pores. The change of the |Δ*D*/Δ*f*| ratio in Figure 6b reflected the tightness degree of lipase adsorbed on the chip. At the beginning, lipase molecules mainly interacted with the surface of the chip, and the interaction was strong. Therefore, the |Δ*D*/Δ*f*| ratio was very low. With the increase of the adsorption mass, the enzyme layers got thicker, and the |Δ*D*/Δ*f*| ratio increased rapidly. After the time point of 10 min, more multilayer adsorption of enzyme molecules enhanced the tightness of the layers, and the |Δ*D*/Δ*f*| ratio then became stable.

From Figure 6c,d, we could see that the enzyme activity increased with the increasing adsorption amount, but the specific activity had little change, which is dramatically different from that of gigaporous particles. For the adsorption on the chip, the effect of pores was completely ruled out. However, for gigaporous particles, though most pores distributed above 100 nm, there were still so many small pores in which the diffusion resistance and the structure change of lipase existed, which could reduce the specific activity of lipase.

## 4. Conclusions

The differences of mesoporous particles, gigaporous particles, and a flat surface for enzyme immobilization were investigated in this study. It was found that the adsorption of enzyme in the mesoporous particles was inclined to be monolayer adsorption that may change the enzyme structure, and the small pores limited the mass transfer of the substrate and the product. These factors reduced the specific activity of lipase. As for the gigaporous particles, in the stage of monolayer adsorption, the interaction between lipase molecules and the pores was weaker than that in the mesopores, so the specific activity was higher. With the increasing amount of enzyme, the occurrence of multilayer adsorption could further promote the maintenance of the enzyme activity. When lipase adsorbed on a chip, the behavior was quite different when excluding the influence of pores, the adsorption mass showed a linear increase, the specific activity had little change during the whole process, and the activity continuously enhanced.

## Figures and Tables

**Table 1:** Structural data of PST-300 and PST-14 microspheres.

Microspheres	Average Pore Size (nm)	Total Pore Surface Area (m^2^/g)	Total Pore Volume (cm^3^/g)	Porosity (%)
PST-300	340.3	14.7	1.9	70.4
PST-14	14.5	738.4	2.7	78.7

## Abbreviations

The following abbreviations are used in this manuscript:

PST	polystyrene
CALB	lipase B from *Candida antarctica*
CLSM	confocal laser scanning microscopy
QCM-D	quartz crystal microbalance with dissipation monitoring
